# Comprehensive evaluation of zeolite/marine alga nanocomposite in the removal of waste dye from industrial wastewater

**DOI:** 10.1038/s41598-023-34094-y

**Published:** 2023-05-18

**Authors:** Ahmed Hamd, Mohamed Shaban, Ghadah M. Al-Senani, Mashael N. Alshabanat, Azza Al-Ghamdi, Asmaa Ragab Dryaz, Sayed A. Ahmed, Refat El-Sayed, N. K. Soliman

**Affiliations:** 1 Basic Science Department, Faculty of Oral and Dental Medicine, Nahda University Beni-Suef (NUB), Beni Suef, Egypt; 2 Department of Physics, Faculty of Science, Islamic University of Madinah, Madinah, 42351 Saudi Arabia; 3 Nanophotonics and Applications Lab, Physics Department, Faculty of Science, Beni-Suef University, Beni Suef, 62514 Egypt; 4 Department of Chemistry, College of Science, Princess Nourah Bint Abdulrahman University, P.O. Box 84428, 11671 Riyadh, Saudi Arabia; 5 Department of Chemistry, College of Science, Imam Abdulrahman Bin Faisal University, P.O. Box 1982, 31441 Dammam, Saudi Arabia; 6 Renewable and Sustainable Energy Unit, Basic and Applied Scientific Research Center (BASRC), Imam Abdulrahman Bin Faisal University, P.O. Box 1982, 31441 Dammam, Saudi Arabia; 7 Department of Chemistry, Faculty of Science, Beni-Suef University, Beni Suef, 62511 Egypt; 8 Basic Science Department, Faculty of Engineering, Nahda University Beni-Suef (NUB), Beni Suef, Egypt; 9 Department of Chemistry, University College in Al-Jamoum, Umm Al-Qura University, Mekka 25376, Saudi Arabia; 10 Chemistry Department, Faculty of Science, Benha University, Benha, Egypt

**Keywords:** Chemistry, Environmental sciences

## Abstract

A systematic study integrating laboratory, analytical, and case study field trial was conducted to figure out the effective adsorbent that could be used for the removal of Congo red (CR) dye from industrial wastewater effluent. The ability of the zeolite (Z) to adsorb CR dye from aqueous solutions was evaluated after it was modified by the *Cystoseira compressa* algae (CC) (Egyptian marine algae). Zeolite, CC algae were combined together in order to form the new composite zeolite/algae composite (ZCC) using wet impregnation technique and then characterized by the aid of different techniques. A noticeable enhancement in the adsorption capacity of newly synthesized ZCC was observed if compared to Z and CC, particularly at low CR concentrations. The batch style experiment was selected to figure out the impact of various experimental conditions on the adsorption behavior of different adsorbents. Moreover, isotherms and kinetics were estimated. According to the experimental results, the newly synthesized ZCC composite might be applied optimistically as an adsorbent for eliminating anionic dye molecules from industrial wastewater at low dye concentration. The dye adsorption on Z and ZCC followed the Langmuir isotherm, while that of CC followed the Freundlich isotherm. The dye adsorption kinetics on ZCC, CC, and Z were agreed with Elovich, intra-particle, and pseudo-second-order kinetic models, correspondingly. Adsorption mechanisms were also assessed using Weber's intraparticle diffusion model. Finally, field tests showed that the newly synthesized sorbent has a 98.5% efficient in eliminating dyes from industrial wastewater, authorizing the foundation for a recent eco-friendly adsorbent that facilitate industrial wastewater reuse.

## Introduction

No one can deny that water is a vital source of life on Earth. Although industrialization and innovation have enhanced mankind’s way of life, they are also the main cause of pollution to clean water resources^1^. Heavy metal, dye, pharmaceutical, and surfactant molecules, personal care items, pesticides, and some other substances are not only daily sources that pollute pure and restricted water resources daily but also have a dangerous effect on all living things^2–6^. Synthetic dyes that are used in a variety of industries, including paper, rubber, textiles, dyes, printing leave, plastics, and cosmetics are the source behind a bulky number of contaminants in the water^2,7^. This huge expansion in dye usage has led to water pollution and environmental problems. These dyes are largely non-degradable, stable, and toxic^2,8^. Dyes cause mutations, respiratory toxicity, chromosomal fractions, and cancer^9^. For example, people exposed to Congo red (CR) dye, will suffer from extreme eye and skin irritation that are relieved within minutes. Besides, consuming CR can lead to stomach irritation, nausea, vomiting, and diarrhea^10^. These pollutants have been removed using a variety of physical, chemical, and biological techniques. This was accomplished by the use of reverse osmosis, coagulation, electrochemical, membrane separation process, dilution, flotation, filtration, and softening techniques^11–13^.

Compared with the above methods, adsorption is one of the most convenient used method because of low-cost, modest, and low-maintenance, and it is straightforward to handle, with smaller quantities of sediment than other methods^14–20^. In past decades, clay minerals, biomass wastes, agricultural residues algae, fly ash, and activated carbon have been used as effective and cheap adsorbents for eliminating dye from wastewater^21–30^. Because there are active function groups, (for example, carboxylic, hydroxyl, amino, carbonyl, phosphates, sulfonic), pollutants attached to the wall of the biomaterials. In addition, zeolite (Z) has traditionally been used for water softening; It is also used in wastewater treatment, catalytic processes, alternative Z production, disinfection purposes, construction, pulp and paper, coatings, membrane separation, refractories, ceramics, and plastics industries^31–33^.

In our work, a comprehensive study including experiments and field tests is carried out to find out the most suitable adsorption condition that effectively removes CR dyes, from industrial wastewater. Under different experimental conditions, the adsorption performances of Z, Cystoseira compressa algae (CC), and zeolite/algae composite (ZCC) for CR dye removal from wastewater were examined. The influence of CC on Zeolite's adsorption capability has been also followed up. CC and Z were nominated for a variety of reasons, including being abundant and low-cost, natural adsorbents. In addition, the expenses of Z, CC, and ZCC regeneration and reuse are lower, which could help make this a viable process. Definitely, the reuse of a low-cost adsorbent helps reduces the residue removal cost. The effects of contact times, initial dye concentrations, temperature, adsorbents doses, and pH values on CR elimination and their adsorption kinetics and isotherms were investigated using batch experiments.

## Materials and methods

### Materials

CR dye was purchased from Sigma Aldrich and dissolved in distilled water. CC algae were delivered in dry form from El-Nile company. Zeolite ore was obtained from El-Nasr Mining Company and used as it is without further modification. Sodium hydroxide granules with a purity of 99.99% and HCl (36%) were purchased from Sigma Aldrich for pH adjustment.

### Preparation of zeolite/CC algae composite (ZCC)

Wet hydrothermal impregnation technique was used to fabricate ZCC composite^34,35^. Here equal weights of zeolite and dry CC algae were combined in small amount of distilled H_2_O and magnetically stirred for 60 min at 500 rpm, followed by 60 min in an ultrasonic bath to form a paste and to attain a homogeneous impregnation of CC in the surface of Z support. The obtained paste was dried in a vacuum oven at 60 °C for 24 h. The Z, CC, and ZCC composite were characterized by Fourier transformer infrared (FTIR) spectrometer, X-ray diffractometer (XRD), and Scanning electronic microscopy (SEM).

### Preparation of the adsorbate

In this work, the adsorbate was chosen to be CR, a common anionic dye. CR is the sodium salt of 3,3′-([1,1′-biphenyl]-4,4′-diyl)bis(4-aminonaphthalene-1-sulfonic acid)with a formula: C_32_H_22_N_6_Na_2_O_6_S_2_ as shown in Fig. S1 (Supplementary data). CR stock solution of concentration of 1000 ppm was prepared by dissolving 1 g of CR dye in 1 L of distilled H_2_O. CR solutions were prepared at various concentrations using the diluting method. The pH was adjusted to 3, 5, 7, and 10 by adding 0.1 M NaOH or 0.1 M HCl solution to the solution.

### Adsorption studies

Four sequences of adsorption experiments were carried out on Z, CC, and ZCC adsorbents under various adsorption conditions. The studied adsorption parameters are starting CR concentration, adsorbent dose, temperature, and the solution’s pH value, Table S1 (Supplementary data). All CR adsorption studies were carried out using batch experiments under a variety of adsorption parameters; adsorption time (up to 480 min), CR starting concentration (5–25 mg/L), pH (3–10), temperature (25–90 °C), and adsorbent dose (0.01–0.05 g per 20 mL of CR solution) with continuous shaking.

The CR solution volume was fixed at 20 mL in each experiment. By tracking the absorption peak, the CR concentration variation was evaluated with a Perkin Elmer Lambda 950 UV/Vis/NIR spectrophotometer. Z, CC and ZCC reusability were investigated for four cycles where 20 mg of each adsorbent was added to 20 mL of the CR dye with initial concentration 10 mg/L and the experiments were conducted for 480 min at fixed conditions of temperature and pH (25 °C and pH7). After each run; the adsorbent was removed from the solution, washed with distilled water and prepared for the next run.

The CR removal % as well as the CR elimination amounts after a period t (q_t_) and at equilibrium (q_e_) were calculated using Eqs. 1 and 2^36,37^.

1 $${\text{CR dye removal}}\; \% =\frac{\left({\mathrm{C}}_{0}-{\mathrm{C}}_{\mathrm{i}}\right)}{{\mathrm{C}}_{0}}\times 100$$

2 $${\mathrm{q}}_{\mathrm{i}}=\left({\mathrm{C}}_{0}-{\mathrm{C}}_{\mathrm{i}}\right)\frac{\mathrm{V}}{\mathrm{m}};\quad \mathrm{i}=\mathrm{e},\mathrm{ t}$$

At which C_o_ is the CR starting concentration in mg/L and C_t_ is the CR concentration after time t (i = t) and C_e_ is the CR concentration at equilibrium (i = e). V is volume in mL, and m is the mass of the adsorbents in mg. The existing data points are the averages of three separate trials.

### Adsorption isotherms

The adsorption isotherms of the fabricated Z, CC, and ZCC nanocomposite for the studied CR were explained using Freundlich, Langmuir, and Temkin models^38–40^. In supplementary data more details regarding the adsorption isotherms equations and their parameters are explained. Equation 3 can be applied to define the Langmuir isotherm's degree of favorability for equilibrium data utilizing the dimensionless separation factor's value (R_L_)^41^.

3 $${\mathrm{R}}_{\mathrm{L}}=\frac{1}{(1+{\mathrm{K}}_{\mathrm{L}}{\mathrm{C}}_{{\max}})}$$

where C_max_ denotes the CR dye maximum initial concentration.

### Adsorption kinetics and mechanisms

Several adsorption mechanisms and kinetic models such as internal particle diffusion, pseudo-first and second-order models, and Elekovech kinetic models are investigated to determine the adsorption mechanisms and kinetics associated with CR adsorption on Z, CC and ZCC^4,5,42–46^. Supplementary data presented more details about adsorption kinetics equations and their parameters. The average value of all adsorption results was measured in triplicate. OriginPro 2018 statistical functions were used to obtain regression coefficient (R^2^) values for various kinetic and isothermal models.

### Field experiments

The newly manufactured adsorbent was evaluated as an effective eco-friendly adsorbent that could be used on a large scale to get rid of industrial waste dye from industrial wastewater. In this regard, wastewater samples containing waste dyes were delivered from a garment dyeing plant in Beni-Suef, Egypt. The wastewater samples obtained were used without further purification or dilution. The best adsorbent system was selected based on experimental results.

## Results and discussion

### Adsorbents characterizations

#### SEM characterization

**Figure 1:**
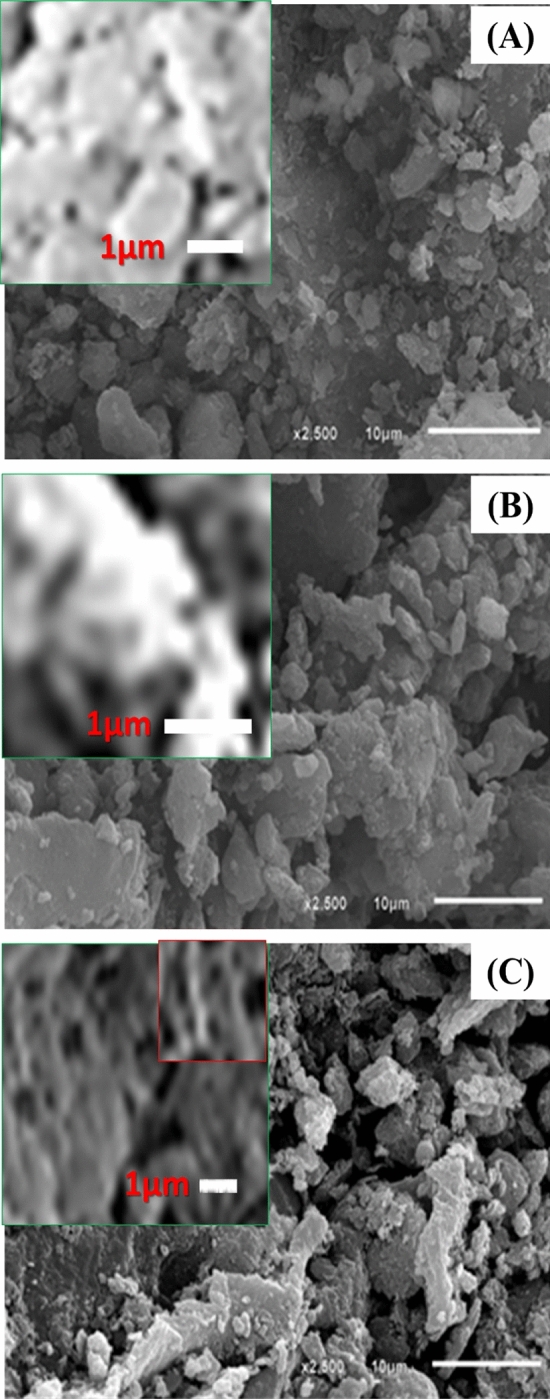
SEM images of (**A**) Z, (**B**) CC, and (**C**) ZCC adsorbents.

Figure 1 shows the SEM images of Z, CC, and ZCC adsorbent. Figure 1A, the SEM image of zeolite, showing flake like structure with different particle sizes and rough surface with porous cavities on the surface which appears clearly in the SEM figures of natural Zeolite. Moreover, the coincidence between SEM and XRD data appears clearly in the crystallinity of zeolite samples revealed in SEM images. SEM images of CC alga, Fig. 1B, reveals the presence of cell wall and different porous structure. The SEM image of CC, reveals a less porous surface that affects its surface area which in turn affects its adsorption capacity. The roughness appearance on CC surface indicates the incorporation of different functional groups, amine, carboxylic groups, and alkyl groups within the pores of the cell wall of CC algae. Finally, modification of the zeolite surface with CC algae, the pores on the zeolite surface are covered with the CC particles as shown in the SEM image of the ZCC composite. These particles are self-assembled to show a rough surface from the agglomerated particles. SEM images of ZCC nanocomposite Fig. 1C confirm the formation of new type of structure as one can see the roughness as well as crystalline structure which indicates the incorporation of CC algae within the structure of zeolite.

#### XRD characterization

**Figure 2:**
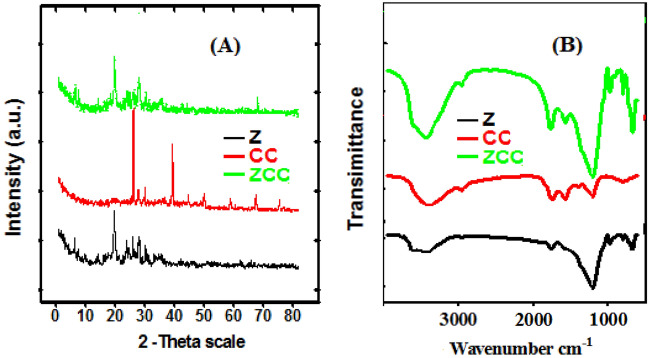
(**A**) XRD and (**B**) FTIR charts of Z, CC, and ZCC adsorbents.

Figure 2A presents the XRD charts of Z, CC, and ZCC adsorbents. In line with other studies findings^47,48^, the zeolite characteristic diffraction peaks appear at 2θ of ~ 9.85°, 22.41°, 26.15°, 26.84°, 28.12°, 30.075° and 32.04°. A d-spacing values of 3.968 Å and 3.173 Å were found for the zeolite major peaks at 22.41° and 28.12°, respectively. The primary peaks in the XRD chart of CC are found at ~ 28.50°, 29.89°, 31.85°, 40.66°, 45.57°, 50.37° and 66.52°. The typical major peaks of ZCC are shown in the XRD chart at around 10.01°, 11.32°, 17.50°, 21.8°, 22.54°, 26.32°, 26.94°, 30.05°, 32.18° and 66.97°. The average crystallite sizes were calculated using the Scherer equation and were found to be 34.4 nm, 57.7 nm and 56.7 nm for Z, CC and ZCC, in order, which is considered a proof for the nanostructure nature of the newly synthesized composite.

#### FT-IR study

Figure 2B shows the FT-IR charts of Z, CC, and ZCC adsorbent. The spectrum showed a broadband at 3432, 3442, and 3452 cm^−1^ which are related to the inner OH stretching^49–51^. For zeolites, the band at 1029 cm^−1^ corresponds to the Si–O vibrational mode, for ZCC this band shifts to 1039 cm^−1^^60^. The Si–O–Al and octahedral aluminum (Al–OH) bands appears at 603 and 919 cm^−1^, respectively^52^. The Si–O–Si bending of zeolite shows a band at 464 cm^−1^, for ZCC this band is shifted to 461 cm^−1^^52^. The metal oxides bands appeared in the region from 400 to 800 cm^−1^^53^.

For the CC algae, the band attributed to the the stretching vibration of amine groups (–NH) appears at 3787 cm^−1^, while that of the hydroxyl group (–OH) of phenolic compounds appears at 3432 cm^−1^. The alkyl groups (–CH) stretching band appears at 2915 cm^−1^, while the –C=O vibration band looks at 1627 cm^−1^. The C–H vibration mode located at 1425 cm^−1^^54,55^. The bands situated around 1019 cm^−1^ refer to the C–O bond or the sulfate group^56^. The N–H stretching vibration mode of amines appears around 3300–3500 cm^−1^. While the O–H stretching mode of a carboxylic group located at 2915 cm^−1^^57^. In line with data obtained from other characterization techniques, both band shift and band disappearance confirms the new ZCC composite formation. Bands shift and assignment are summarized in Table S2.

### Factors affecting the adsorption process

#### Effect of initial dye concentration

**Figure 3:**
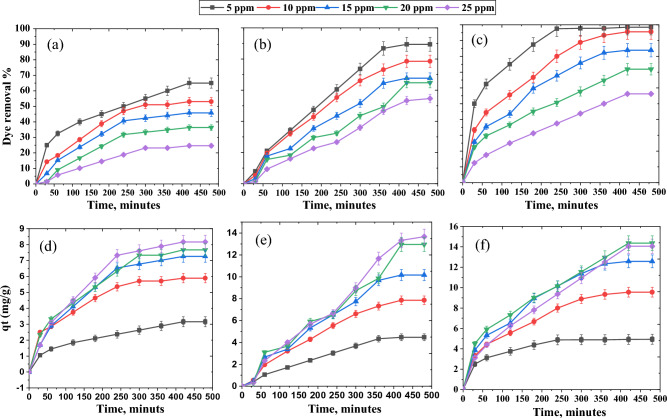
Impact of CR starting concentration and reaction time on the removal% and the amount of CR adsorption at 25 °C, pH 7, and 20 mg of (**a**,**d**) Z, (**b**,**e**) CC, and (**c**,**f**) ZCC.

The amount of CR removed by adsorption is highly dependent on the starting CR concentrations. The variations in the elimination percent and the amount of CR adsorbed using Z, CC, and ZCC adsorbents at different initial concentrations versus time were demonstrated in Fig. 3a–f. Throughout the first stage of the adsorption process, the dye elimination percentages and the adsorption capacities are very high. Their growth slows down until equilibrium is reached. The presence of a large number of active sites found on the surface of the adsorbent can explain the rapid rate of removal at the beginning of the reaction.

As the time procced, the active sites are completely occupied by CR molecules^58^. This results in repulsive interactions between CR molecules in the attractive regions of the adsorbent and those in the bulk liquid phase^37^. Consequently, the fraction of CR released decreases as the initial CR concentration increases. Over all the tested CR concentrations, the composite, ZCC, shown the highest adsorption efficiency, where ZCC > CC > Z shows the CR removal % in the previous order. The amount of CR adsorbed increases as the starting CR concentration rises. This could be elucidated to the concentration gradient grows as the initial CR concentration rises, as shown in Fig. 3d–f. Hence, the driving force grows, which is the main reason for overcoming the barrier for mass transfer between Z, CC, and ZCC adsorbents and CR adsorbates^3,59^. For CR with starting concentrations of 25, 20, 15, 10, and 5 mg/L, the highest adsorption capacities of ZCC were reported to be 14.06, 14.36, 12.58, 9.50, and 4.90 mg/g, respectively. At pH 7 and 25 °C for CR with starting concentrations of 25, 20, 15, 10, and 5 mg/L, maximal adsorption capacities were 13.60, 12.90, 10.16, 7.80, and 4.40 mg/g for CC and 3.10, 5.90, 7.25, 7.60, and 8.10 mg/g for Z. The results indicated that the addition of CC to Z is a practicable method to improve the CR uptake routine of Z.

#### Adsorbent dosage effect

**Figure 4:**
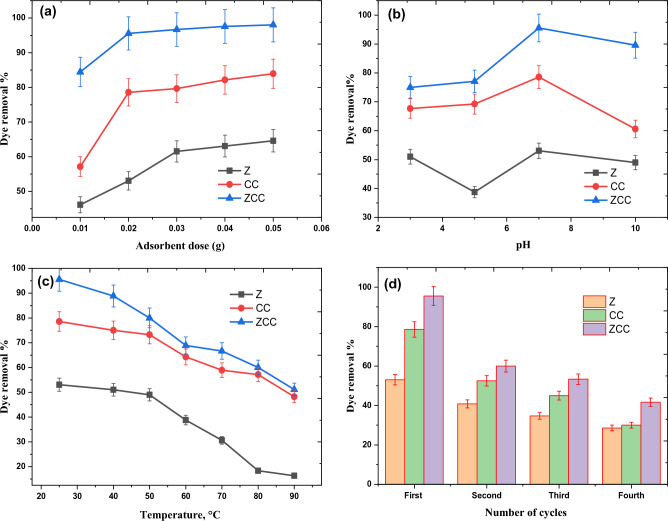
Effect of (**a**) adsorbent dose, (**b**) starting pH, (**c**) adsorption temperature, and (D) reusability test on the removal% of 10 mg/L CR solutions by Z, CC, and ZCC.

At the optimal adsorbent dosage for maximum efficiency, The influence of the adsorbent dosage on the CR removal% was estimated with respect to adsorption cost. This is shown graphically in Fig. 4a, where the sorbent dosage varied from 0.01 to 0.05 g. From Fig. 4a, we notice that, for all adsorbents, the dye removal% increases by rising the sorbent dosage from 0.01 to 0.05 g; in the case of Z adsorbent, it increased from 46.15 to 64.61%, increased from 57.14 to 83.93% for CC adsorbent and from 84.44 to 98.00% for ZCC adsorbent. Increasing the number of hot spots by increasing the adsorbent mass could be the reason for which this observation could be accredited^3,4,37^. It was observed that significant jumps in removal occurred as the adsorbent amount of ZCC increased from 0.01 to 0.02 g and that of Z increased from 0.01 to 0.03 g. In the case of ZCC and Z, the variation of CR % removal was slightly reduced by increasing the sorbent dose above 0.02 g and 0.03 g, respectively. This may be due to the “shielding effect” that occurs when the amount of adsorbent increases. Due to the increase of adsorbent amount and the reduction of spaces between them, a dense layer is formed on the surface of the adsorbent. The active site is hidden from the CR molecule by the formation of a dense layer. Furthermore, CR molecules compete for a limited number of accessible active sites due to the overlap of Z and ZCC. Aggregation or agglomeration at high doses of Z and ZCC increases the diffusion path length of CR adsorption, thereby reducing the adsorption rate^43,60–62^.

#### Influence of pH

pH is considered one of the most essential parameters influencing the dye removal capacity of adsorbent in wastewater. The adsorption efficacy is affected by the pH of the solution, as the pH changes affect the ionization degree of the adsorbate molecules as well as the surface characteristics of the adsorbent^63^. In the pH range between pH 3 and pH 10, the pH influence on the CR removal percentage by Z, CC and ZCC adsorbents was studied as shown in Fig. 4b. A removal percentages of ~ 51.02%, 38.78%, 53.06%, and 48.98% were recorded for Z adsorbent at pH values of 3, 5, 7, and 10, in order, using 20 mL of 10 mg/L of CR solution and adsorbent dosage of 0.02 g. At the same previously mentioned conditions, a 67.69, 69.23%, 78.57%, and 60.60% removal percentages were observed for the CC adsorbent while ZCC adsorbent represents removal percentages 75%, 77.08%, 95.55%, and 89.58% at pH values of 3, 5, 7, and 10, respectively. Figure 4b shows that when the pH of the solution is 7, the CR adsorption capacity of Z, CC, and ZCC reached their maximum limit. This may be due to the interaction between the CR and adsorbent is more prominent than the interaction between the adsorbent with OH^−^ ions in the solution^64^. At lower pH levels, the positive charge on the solution interface growths, and the Z, CC, and ZCC surfaces appear to be positively charged. However, due to the protonation of the CR molecules, CR in the solution tends to be neutral. This situation leads to a reduction in the adsorption of anionic CR as presented at pH 5^65^. With increasing pH value, the positive charge on the solution interface reduces and CR becomes negatively charged by the interaction with OH^-^ ions. Hence, positively charged Z, CC, and ZCC have a reasonable interaction with negatively charged CR molecules or with OH^-^ ions. Therefore, when pH exceeds7, CR adsorption decreases^73^.

#### Influence of temperature

The temperature effect is refered as a considerable physicochemical factor in the adsorption study, due to the variation caused in the adsorption capacities of the adsorbents^66^. A temperature series was applied at temperature ranges 25, 40, 50, 60, 70, 80, and 90 °C, all data are presented in Fig. 4c. A reverse relationship was noticed between temperature and dye removal percent. This relationship could be ascribed by the fact that adsorption force brokendown upon exposure to temperature, this force is responsible for dye molecule adsorption on the adsorbent surface^75^. Moreover, damaging and weakening of the active sites between the adsorbent's active binding sites and the adsorbate species^3,76,77^. Consequently, the optimal temperature for CR adsorption onto all tested adsorbents was selected to be located between 25 and 30 °C. With the temperature, the percentage of CR removal decreases, indicating exothermic adsorption process.

#### Reusability test

All adsorbents under investigation (Z, CC, and ZCC) were subjected to a reusability experiment which was repeated for 4 cycles using identical adsorbent doses. The data illustrated in Fig. 4d revealed that; the removal percentage of all adsorbents under investigation varied throughout the four adsorption cycles. A noticeable decreaseing in the removal percent for Z adsorbent was recorded 53.06%, 40.82%, 34.69%, and 28.57%, in the 1st, 2nd, 3rd and 4th cycles, within the same order. While for CC, the dye removal percent revealed a sharp decreased from 78.57% in the 1st cycle to 30% in the 4th cycle. Finally, the ZCC nanoadsorbent, the dye removal percent was dropped from 95.55% in the 1st cycle to 41.67% in the 4th cycle. This behavior could be explained by the fact that CR dye molecules upon reusability agglomorise on the surfaces of Z, CC, and ZCC adsorbents and hence hides the adsorbent surfaces and pores from the dissolved CR molecules, resulting in a loss in adsorption capacity^67^.

### Adsorption isotherms

**Figure 5:**
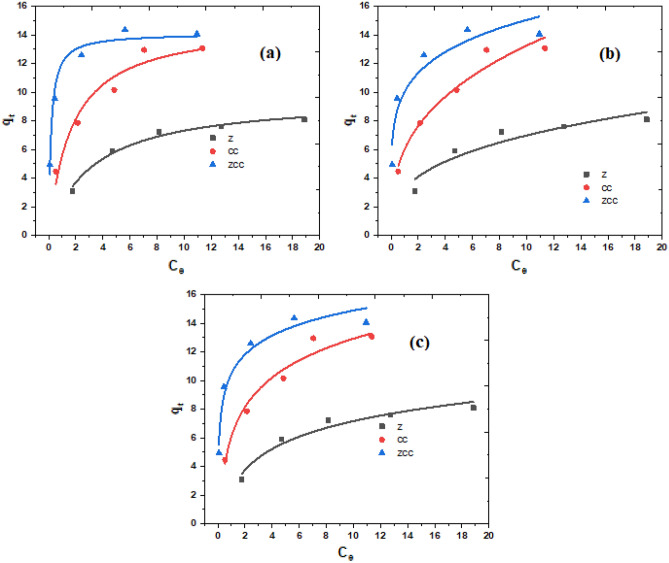
Plots showing the adsorption isotherms of (**a**) Langmuir, (**b**) Freundlich, and (**c**) Temkin for the adsorption of CR using Z, CC, and ZCC at 25 °C and pH 7.

All data were fitted to Langmuir, Freundlich, and Temkin models using the statistical significance of correlation coefficient (R^2^) for nonlinear fitting of Ce versus q_e_. Table 1 presents all values related to of Q_o_, K_L_, K_F_, n, K_T_, B, and R^2^, these values were computed from the nonlinear fitting of the plots in Fig. 5. From the data revealed in Table, CR adsorption on Z and ZCC adsorbents follows the Langmuir model with the best R^2^ value. Consequently, a multilayer adsorption of CR molecules occured at the active sites on the surface of the adsorbent under investigation. At these active sites there are unequally available heterogeneous sites, each with varied adsorption energy and interacting molecules. The values of R^2^ obtained from the fitting to Langmuir isotherms in case of Z and ZCC adsorbents were 0.9837 and 0.9737 at 25 °C, respectively. The R_L_ value is less than unity, which indicates that the adsorption of CR in that studied case is valuable^68^.

**Table 1:** Isotherms parameters for CR adsorption on Z, CC, and ZCC.

Adsorbent	Constant			
	R^2^	R_L_	K_L_ (L/mg)	Q_o_ (mg/g)
Langmuir isotherm				
ZCC	0.9737	0.0068	5.781	14.11
CC	0.9479	0.062	0.599	14.91
Z	0.9837	0.114	0.309	9.67

Adsorbent	Constant			
	R^2^	K_f_	n	
Freundlich isotherm				
ZCC	0.9099	10.03	5.69	
CC	0.9584	6.02	2.92	
Z	0.8960	3.25	3.02	

Adsorbent	Constant			
	R^2^	K_T_ (L/mole)	B (J/mol)	
Temkin isotherm				
ZCC	0.9640	239.68	1.91	
CC	0.9540	7.81	2.69	
Z	0.9569	2.93	2.12

On the other hand, the adsorption isotherm of CR onto CC followed the Freundlich model. Consequently, a single layer adsorption process occued on the active sites of the CC adsorbent, and no reaction occurred between the adsorbed CR molecules. The R^2^ value was recored to be 0.9584 for the Freundlich isotherms of CC adsorbent, also the R_L_ value is less than unity, indicating that the CR adsorption in the investigated situation has considerable valuability^68^. Moreover, The values of (1/n) in the Freundlich isotherm model in the adsorption process of CR dye on the CC adsorbent were less than unity, which indicaded that adsorption was valuble, and heterogenisity of the surface, with fewer interactions between adsorbed ions. Meanwhile, It refers to the CR adsorption occured by multi-molecular and multi-anchorage adsorption mechanisms, respectively^69,70^.

### Adsorption kinetics

**Figure 6:**
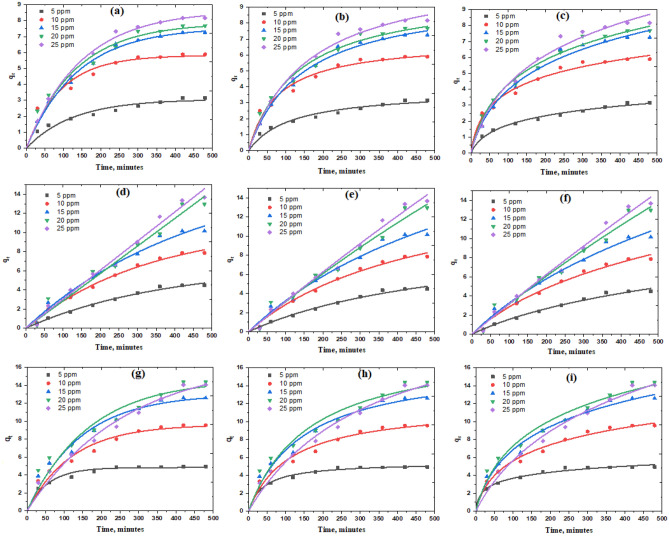
(**a**,**d** and **g**) Pseudo-first-order, (**b**,**e** and **h**) Pseudo-second-order, and (**c**, **f** and **i**) Elovich sorption kinetics of CR at 25 °C and pH 7 using 0.020 g of Z, CC, and ZCC, respectively.

In order to study the most favourable kinetic model, the process of the adsorption of CR on the surface of Z, CC, and ZCC adsorbents was followed at varying initial dye concentrations. t vs qt was used and plotted in Fig. 6 in order to show the nonlinear forms of the first-order, second-order, and Elovich kinetic models. After that, each of kinetics and statistical parameters; k_1_, k_2_, q_e_, β, α, and R^2^; were obtained and all the data were presented in Table 2. The nonlinear regression values of the studied concentration range and examined models which presented in Table 2 indicated that adsorption of CR onto Z surface was fitted by the first-order kinetics at higher concentrations (15, 20, and 25 ppm). Which was confirmed and supported by the good approximation between the estimated q_e_ and experimental q_exp_. Adsorption of CR onto Z surface was fitted by Elovich kinetics at CR concentrations of 5 and 10 ppm indicating that adsorbent surfaces are not energetically homogeneous. While, in case of ZCC, the Elovich kinetics found to be more suitable indicating that adsorbent surfaces are not energetically homogeneous which also confirmed by the higher R^2^ values^71^. Finally, the adsorption of CR molecules onto the surface of CC adsorbent follows first-order kinetics at lower concentration, while it follows Elovich kinetics at lower concentration.

**Table 2:** Kinetic model parameters for CR dye adsorption on Z, CC, and ZCC.

Adsorbents	Conc. (ppm)	First order				Second order				Elovich kinetic model		
		q_e_ exp	q_e calc_	k_1_	R^2^	q_e_ exp	q_e calc_	k_2_	R^2^	β (g/mg)	α (mg/min)	R^2^
Z	5	3.1	3.06	0.0079	0.9518	3.1	3.76	0.0023	0.9741	1.06	0.052	0.9892
	10	5.9	5.82	0.0107	0.9654	5.9	6.91	0.0018	0.9807	0.64	0.166	0.9867
	15	7.25	7.56	0.0073	0.9948	7.25	9.80	7.020E-4	0.9934	0.36	0.089	0.9885
	20	7.6	7.85	0.0075	0.9988	7.6	9.90	7.724E-4	0.9865	0.38	0.109	0.9900
	25	8.1	8.63	0.0068	0.9956	8.1	11.37	5.444E-4	0.9927	0.30	0.089	0.9871
CC	5	4.47	6.50	0.0026	0.9929	4.47	10.43	1.669E-4	0.9896	0.24	0.018	0.9884
	10	7.85	10.60	0.0031	0.9933	7.85	16.59	1.244E-4	0.9919	0.15	0.036	0.9904
	15	10.16	16.40	0.0022	0.9848	10.16	27.18	5.003E-5	0.9841	0.09	0.038	0.9835
	20	12.95	17.28	3.902E-5	0.9730	12.95	90.98	3.931E-6	0.9767	0.02	0.033	0.9768
	25	13.66	18.40	1.643E-5	0.9862	13.66	183.26	9.638E-7	0.9872	0.01	0.032	0.9877
ZCC	5	4.93	4.82	0.0177	0.9694	4.93	*5.43*	*0.0044*	0.9893	1.02	0.416	0.9897
	10	9.55	9.60	0.0083	0.9631	9.55	11.82	7.652E-4	0.9789	0.34	0.168	0.9883
	15	12.58	13.02	0.0069	0.9731	12.58	16.61	4.173E-4	0.9818	0.22	0.163	0.9873
	20	14.36	14.66	0.0060	0.9440	14.36	18.71	3.249E-4	0.9616	0.03	0.164	0.9773
	25	14.06	16.81	0.0037	0.9781	14.06	24.04	1.230E-4	0.9822	0.13	0.083	0.9862

Significant values are in [italics].

### Sorption mechanism

Weber's Intra-particle diffusions was found to be more favourable in the fitting of the experimental findings in order to better understand the mechanisms and rate-controlling processes that impact directly on adsorption kinetics. The nonlinear fitting of q_t_ versus Ce, Fig. S2 (Supplementary data), supports the applicability of the intra-particle diffusion model's. Meanwhile, each of slopes and intercepts of the plots are utilized to compute ***K***_***3***_ and ***I*** value, Table 3. The values of ***I*** ≠ 0 indicated that the intra-particle model may not be the only rate-controlling process in identifying the adsorption process' kinetics^72^. Moreover, the boundary layer effect is clearly reflected by the intercept obtained in Fig. S2. The relation is a reverse on as bigger the intercept, corresponding to more surface adsorption contributes to the rate control step^72^.

**Table 3:** Intra-particle diffusion parameters at various CR concentrations.

Adsorbents	Conc. (ppm)	Intraparticle diffusion kinetic model		
		I	k_3_ (mg/g min^1/2^)	R^2^
Z	5	0.197	0.141	0.9889
	10	0.693	0.268	0.9508
	15	0.130	0.360	0.9703
	20	0.337	0.366	0.9808
	25	0.006	0.412	0.9697
CC	5	− 0.564	0.236	0.9658
	10	− 0.940	0.414	0.9708
	15	− 1.418	0.533	0.9570
	20	− 2.050	0.640	0.9286
	25	− 2.472	0.693	0.9264
ZCC	5	1.051	0.210	0.8739
	10	0.656	0.445	0.9792
	15	0.438	0.601	0.9851
	20	0.411	0.652	0.9929
	25	− 0.622	0.673	0.9896

### Batch experiments and comparison with other adsorbents

Depending on the experimental findings obtained during laboratory tests, well-selected optimized parameters for the newly-synthesized ZCC adsorbent were applied in a real field experiment in order to find out the applicability of our new adsorbent in the real industrial process. The parameters including 0.02 g of the adsorbent, room temperature, a non-changed pH of the wastewater containing waste dye, and the contact time was adjusted at 420 min. The industrial wastewater was subjected to scanning of wavelengths which revealed the presence of different wavelengths corresponding to different dyes. After the experiments were reached to a completion, absorbance was recorded using the same scanning device to estimate the removal percent of the dyes from the industrial wastewater. The promising data revealed that the ZCC nanoadsorbent succeded to catch up the different dyes from industrial wastewater with a 98.5% efficiency, which confirmed the foundation of new environmentally benign adsorbents that could be applied to reuse the industrial wastewater.

Table 4 compares the adsorption capacity, q_m_, and dye removal percent of different studied adsorbents reported in the past work with those of Z, CC, and ZCC for CR dye adsorption. It appears that q_m_ values vary broadly for different adsorbents^73–77^. The results stated that Z, CC, and ZCC displayed reasonable capacities for CR dye adsorption from aqueous solution relative to other adsorbent materials^73–77^.

**Table 4:** Comparison of the optimized conditions, removal%, and adsorption capacity at different CR adsorbents relative to our Z, CC, and ZCC nanoadsorbents.

Adsorbent	Conditions	Adsorption capacity (mg/g)	Removal %	References
Surfactant Modified Zeolite A Synthesized from Fly Ash SMZA	Time: 60 min Concentration: 5-25 mg/L pH: 7 Temperature: 25 °C Adsorbent dose: 10 mg	23.99	83.89%	^73^
Porphyrayezoensis Ueda (red alga)	Time: 600 min Concentration: 80 mg/L pH: 8 Temperature: 25 °C Adsorbent dose: 5 g	71.46	–	^74^
CTAB modified pumice (SMP)	Time: 1440 min Concentration: 10 g/L pH: 8 Temperature: 25 °C Adsorbent dosage: 140 g/L	27.32 (linear form) 18.81 (non-linear form)	98.45%	^75^
Natural pumice (NP)	Time: 360 min Concentration: 10 g/L pH: 8 Temperature: 25 °C Adsorbent dosage: 140 g/l	3.87 (linear form) 4.42 (non-linear form)	98.11%	
Alternanthera bettzichiana Plant powder (ABPP)	Time: 130 min Concentration: 10 mg/L pH: 5 Temperature: 25 °C Adsorbent dose: 2 g/L	14.67	80.5%	^76^
Soil	Time: 40 min Concentration: 50 mg/L pH: 6.8–6.9 Temperature: 30 °C Adsorbent dosage: 2.5 g	8.65	94%	^77^
Z	Time: 420 min Concentration: 20 mg/L pH: 7 Temperature: 25 °C Adsorbent dose: 20 mg	8.1	65.00%	This work
CC		13.67	89.47%	
ZCC		14.06	98.50%	

### Hypothesis for organic adsorption of dye over different studied adsorbents

**Figure 7:**
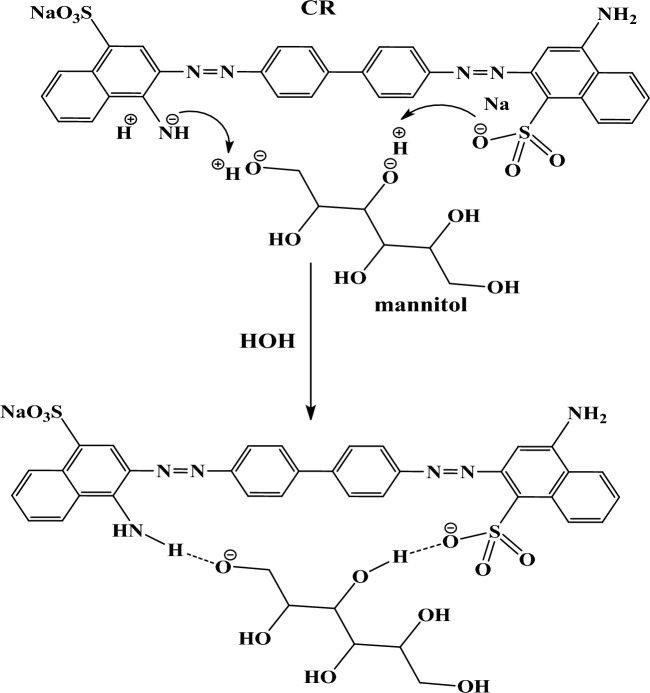
Adsorption mechanism of congo red dye on CC.

**Figure 8:**
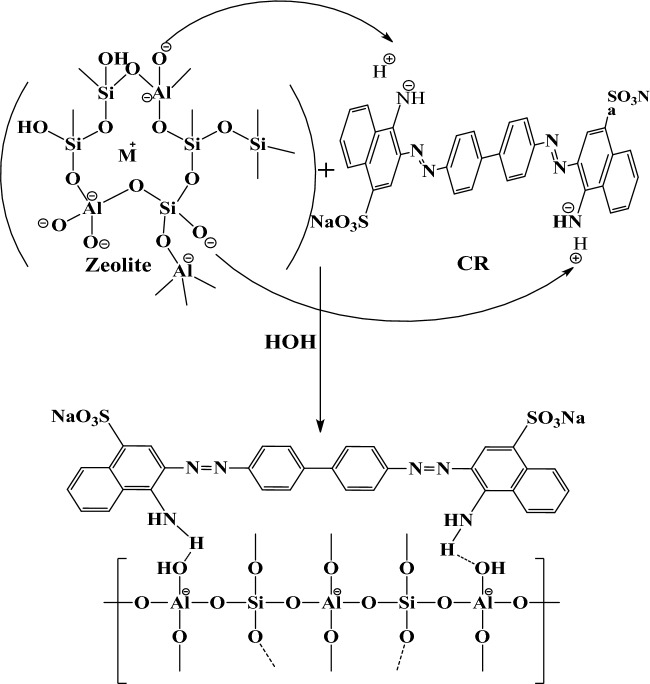
Adsorption mechanism of congo red dye on Z.

**Figure 9:**
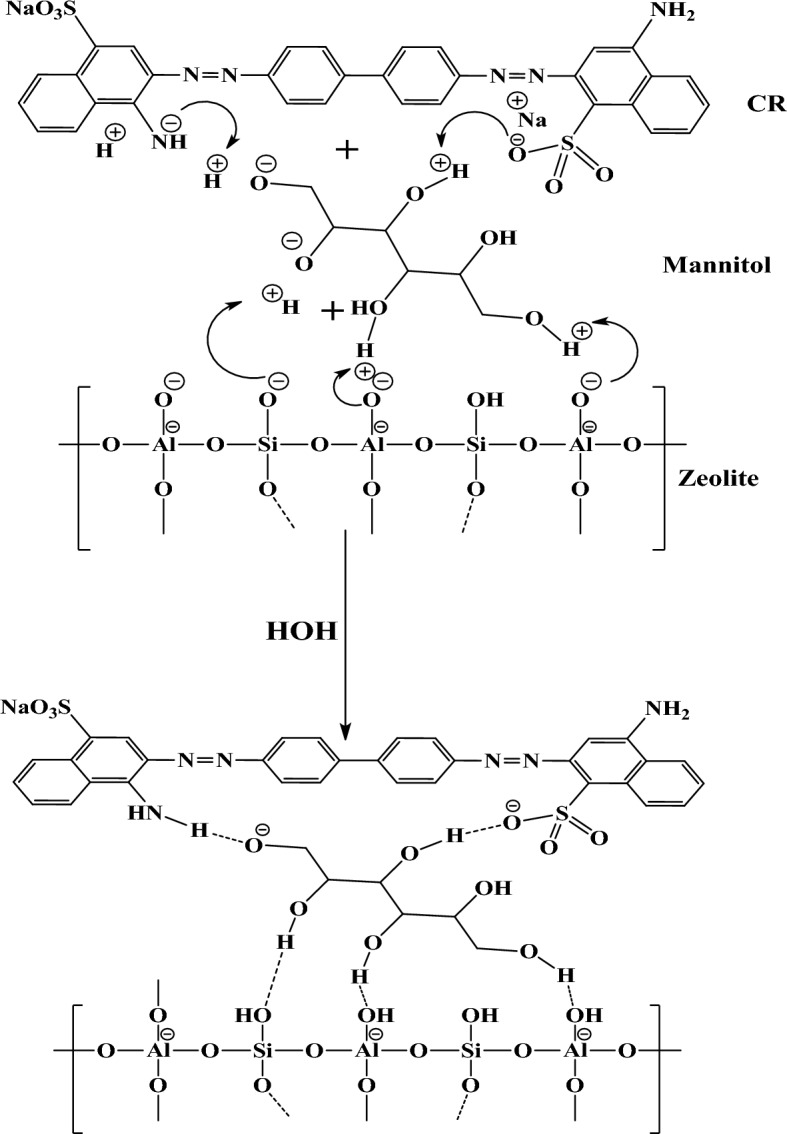
Adsorption mechanism of congo red dye on ZCC Composite.

Figures 7, 8 and 9 reveal interactions between zeolite, Cystoseira compressa, and ZCC composite surface and CR dye molecules. These interactions summarized in hydrogen bonds between oxygen and amine group in CR dye molecule as well as electrostatic interaction between negative and positive charges. This hypothesis confirmed by IR results.

## Conclusions

A hydrothermal technique was selected for the preparation of of novel alga/zeolite composite ZCC from Z and CC. In order to remove CR dye from industrial wastewater, ZCC was introduced as a novel adsorbent at various conditions. The experimental results revealed that the lower the initial CR concentration, the greater the removal percent of CR to be improved also the removal rate was high during the adsorption experiment's early stages. The removal% increased with increasing Z, CC, and ZCC dosage from 0.01 to 0.05 g and it highly affected by temperature. For all adsorbents, the CR removal% increased with changing the initial pH from 3 to 10 and the supreme adsorption occurs at pH 7. The reducibility test for Z, CC, and ZCC adsorbents showed that all the adsorbents were not favored for reuse for the CR removal. The isotherms of CR adsorption onto Z, CC, and ZCC show that Z and ZCC adsorbents track the Langmuir isotherm models while CC follows the Freundlich isotherm models. Moreover, the CR adsorption onto Z was well-fitted to the second-order kinetics, while CC follows the intra-particle kinetics models. Furthermore, the CR adsorption onto the surface of ZCC was well fitted with Elovich kinetics models. More optimistic, the field experiments showed promising results as the ZCC nanoadsorbent succeeded to catch up the different dyes from industrial wastewater with a 98.5% efficiency, which confirmed the new born of novel environmentally benign adsorbents that could be applied to reuse the industrial wastewater. Finally, the supposed mechanisms for the adsorption of CR dye over adsorbents under investigation were in line with the data obtained from the IR charts and all the interactions summarized in hydrogen bonds between oxygen and amine group in CR dye molecule as well as electrostatic interaction between negative and positive charges.

## Supplementary Information

Supplementary Information.

## Data Availability

The datasets used and/or analysed during the current study available from the corresponding author on reasonable request.
