# Supplementary material for: Comprehensive evaluation of zeolite/marine alga nanocomposite in the removal of waste dye from industrial wastewater
Source: Sci Rep. 2023 May 18;13:8082. doi: 10.1038/s41598-023-34094-y (PMC10195818; doi:10.1038/s41598-023-34094-y)
Supplement: Supplementary file 1 — Supplementary Information. [file 41598_2023_34094_MOESM1_ESM.docx]

**Comprehensive evaluation of Zeolite/Marine alga nanocomposite in the removal of waste dye from industrial wastewater**

**Ahmed Hamd ^1^, Mohamed Shaban^2,3^,** **Ghadah M. Al-Senani^4^, Mashael N. Alshabanat^4^, Azza Al-Ghamdi^5,6^, Asmaa Ragab Dryaz^7^, Sayed A. Ahmed^7^, ,** **Refat El-Sayed ^8, 9^**^,^**, N.K. Soliman ^1*^**

^1^ Basic Science Department, Faculty of Oral and Dental Medicine, Nahda University Beni-Suef (NUB), Beni Suef, Egypt.

^2^Department of Physics, Faculty of Science, Islamic University of Madinah, Madinah, 42351, Saudi Arabia.

^3^ Nanophotonics and Applications Lab, Physics Department, Faculty of Science, Beni-Suef University, Beni Suef 62514, Egypt.

^4^ Department of Chemistry, College of Science, Princess Nourah bint Abdulrahman University, P.O. Box 84428, Riyadh 11671, Saudi Arabia

^5^Department of Chemistry, College of Science, Imam Abdulrahman Bin Faisal University, P.O. Box 1982, Dammam 31441, Saudi Arabia.

^6^Basic & Applied Scientific Research Center (BASRC), Renewable and Sustainable Energy Unit, Imam Abdulrahman Bin Faisal University, P.O. Box 1982, Dammam 31441

^7^Department of Chemistry, Faculty of Science, Beni-Suef University, Beni Suef 62511, Egypt

^8^Chemistry Department, Faculty of Applied Sciences, Umm Al-Qura University,Mecca, Saudi Arabia.

^9^ Chemistry Department, Faculty of Science, Benha University, Benha, Egypt;

***corresponding author: nofal.khamis@nub.edu.eg**

1. **Adsorption isotherms**

The reaction isotherms of the developed Z, CC, and ZCC nanocomposite for the tested CR were explained using Langmuir, Freundlich, and Tempkin isotherms. Equations1, 2, and 3 represent the three models, respectively [38-41]:

$qe= \frac{Q_{o}K_{L}C_{e}}{{1+K}_{L}C_{e}}$ (1)

$q_{e}= K_{F}$C_e_ ^1/n^ (2)

$q_{e}= B \mathrm{LnK}_{T}C_{e}$ (3)

Here, Q_o_ is the maximum amount of adsorbed CR by Z, CC, and ZCC adsorbents (mg/g); K_L_, K_F,_ and K_T_ indicate the Langmuir, Freundlich, and Tempkin constants, respectively. B(=RT/b) is a constant related to the adsorbed heat, n is the adsorption density, T is the absolute temperature, and R is the universal gas constant.

1. **Adsorption kinetics and mechanism**

Different adsorption mechanisms and kinetics models such as intra-particle diffusion, pseudo-first-order,pseudo-second-order, and Elovich kinetic models are applied for identifying the adsorption mechanisms and kinetic models that matched the CR adsorption onto Z, CC, and ZCC nanoadsorbents.

Equations 4 to 7 are used to represent the pseudo-first-order, pseudo-second-order, simple Elovich kinetic, and Intra-particle diffusion models, respectively [4, 43-48].

q_t_ = q_e_ (1 – e^k1 t^) (4)

$qt = \frac{k_{2}{t q}_{e}^{2}}{1+k_{2}{t q}_{e}}$ (5)

$q_{t} = \frac{1}{\beta}ln( \alpha\beta t+1)$ (6)

$q_{t} = k_{3}t^{\frac{1}{2}}+I$ (7)

Where k_1_, k_2_, and $k_{3}$ are the pseudo-first-order, pseudo-second-order, and intra-particle propagation models' rate constants. *I* refers to a constant related to the boundary thickness. α denotes the starting adsorption rate (mg/min) and β signifies the extent of surface treatment (g/mg).

Table S1. Conditions of experimental adsorption tests.

Table S1. Conditions of experimental adsorption tests.

| **Series** | **Dye concentration, ppm** | **Z, CC and ZCC weight, g** | **Temperature, ^o^C** | **pH value** |
| --- | --- | --- | --- | --- |
| **1** | 5, 10, 15, 20 and 25 | 0.02 | 25 | 7 |
| **2** | 10 | 0.01, 0.02, 0.03, 0.04 and 0.05 | 25 | 7 |
| **3** | 10 | 0.02 | 25, 40, 50, 60,70, 80 and 90 | 7 |
| **4** | 10 | 0.02 | 25 | 3, 4, 5, 7, 8 and 10 |

Table S2. Characteristic wavenumbers and function groups of FTIR bands for Z, CC, and ZCC adsorbents.

| **FT-IR peaks (cm^-1^)** | | | **Assignment** | **References** |
| --- | --- | --- | --- | --- |
| **Z** | **CC** | **ZCC** |  |  |
| - | 3787 | 3300 – 3500 | amine group (-NH) stretching | [58] |
| 3452, 3432, and 3442 | 3432 | 2915 | hydroxyl group (-OH) | [51-53] |
| - | 2915 | 1425 | (-CH) group | .[23, 56] |
| - | 1627 | 1019 | (-C=O) group | [57] |
| 1029 | - | 1039 | Si-O-Al | [59] |
| 464 | - | 461 | Si-O-Si bending | [60] |
| 400 – 800 | - | 400 - 800 | metal oxides | [55] |


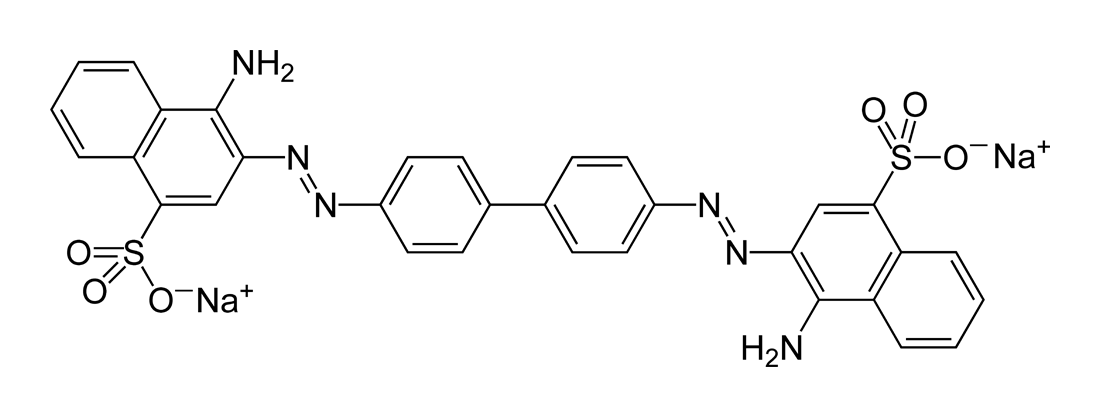


**Fig. S1. Structure of Congo red
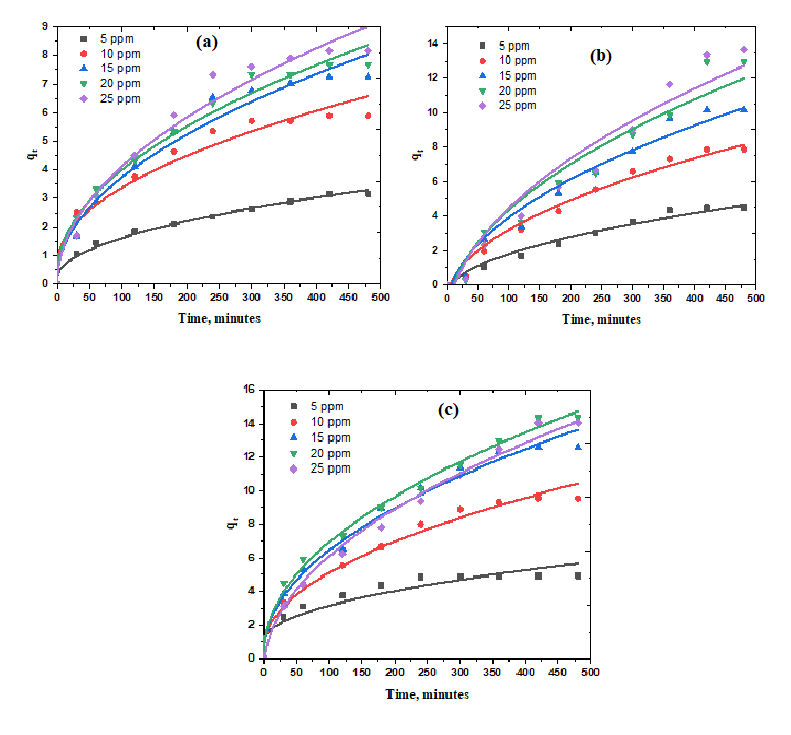
**Figure S2. Intra-particle sorption kinetics of CR dye at 25 ^°^C and pH 7 by 20 mg of (A) Z, (B) CC, and (C) ZCC.
